# Risk Threshold and Assessment of Chloramphenicol Antibiotics in Sediment in the Fenhe River Basin, China

**DOI:** 10.3390/toxics11070570

**Published:** 2023-06-30

**Authors:** Linfang Wang, Dexuan Dang, Leiping Cao, Huiyan Wang, Ruimin Liu

**Affiliations:** 1 Shanxi Key Laboratory of Sorghum Genetic and Germplasm Innovation, Sorghum Research Institute, Shanxi Agricultural University, Jinzhong 030600, China; 2 State Key Laboratory of Water Environment Simulation, School of Environment, Beijing Normal University, Beijing 100875, China

**Keywords:** chloramphenicol antibiotics (CAs), sediment, Equilibrium Partitioning approach (EqP), predicted no-effect concentration (PNEC), risk threshold

## Abstract

Chloramphenicol antibiotics (CAs) are broad-spectrum antibiotics which are widely used in the prevention and treatment of infectious diseases in livestock and poultry breeding. However, overused CAs can enter the watershed and eventually enter the sediment. Antibiotics in sediment can cause secondary pollution through disturbance and suspension. In this study, taking the Fenhe River Basin as the research area, the risk of CAs in sediment were assessed by collecting sediment samples. The results showed that CAs were detected in all sediment samples of the Fenhe River Basin. The mean concentration of CAs was 79.1 μg/kg, and the concentration of thiamphenicol (THI) was dominant, which was up to 58.3 μg/kg. Temporally, there are great differences in different seasons; the concentration of CAs was higher in winter than that in summer, up to 4.79–174 times. Spatially, the mean concentration of CAs in midstream was 83.5 μg/kg, which was higher than that in the upstream and downstream. The concentration of CAs in tributaries were generally higher than that in the main stream, and the mean concentration of tributaries was 1.1 times that of the main stream. CAs in S2 (Lanhe River) was the most prominent among all sample sites; the concentration of CAs was 190.8 μg/kg. The risk threshold of CAs in the sediment was calculated using the Equilibrium Partitioning approach (EqP), based on the distribution coefficient (*Kp*) and the predicted no-effect concentration (PNEC) in the water, and the values were 0.091–1.44 mg/kg. Based on the risk threshold, the ecological risk of the CAs in sediment was assessed using risk quotients (*RQ*). The results showed that the Chloramphenicol (CHL) was the most prominent in the Fenhe River Basin, and the proportion of medium-risk areas reached 21.7%, while all the other areas showed low risk. Secondly, the proportion of medium-risk areas was 17.4% for THI, and all the other areas showed low risk. The risk for Florfenicol (FF) was least among all CAs, and the proportion of low-risk areas was only 8.7%, while all the other areas were of insignificant risk.

## 1. Introduction

Chloramphenicol antibiotics (CAs) belong to the wider group of amidol drugs and broad-spectrum antibiotics, which have good inhibitory effects on most gram-negative bacteria and gram-positive bacteria [1]. With the widespread use of antibiotics, people’s lives are improved, while excessive use is very common [2]. A large number of antibiotics can enter the watershed through sewage treatment plants, surface runoff, seepage, and other ways [3,4]. The residual antibiotics in the watershed can accumulate for a long time, causing damage to the microbial community in the water and affecting the aquatic organisms [5,6]. At the same time, antibiotics can destroy the ecosystem and enter the human body through food transmission, which could cause high risk for both ecology and human health [7,8]. Therefore, it is very important to study the CAs in the watershed.

After being discharged into the watershed, CAs can further enter the sediment through dissolution and adsorption [9]. Particles in the sediment can bind CAs firmly, avoiding degradation [10]. CAs in the sediment will enter the water again through disturbance and suspension under specific conditions, resulting in secondary pollution [11,12]. So, CAs in sediments is one of the main sources of pollution in watershed [13,14]. In recent years, researchers have gradually begun to pay attention to the CAs in sediments. Studies showed that the concentration of CAs in sediments in China was higher than that of coastal India, as well as that in the inland rivers of Pakistan and Ethiopia [15,16]. So, the study of CAs in sediments is also extremely critical.

At present, there are many studies about the CA risk threshold of water [17,18]. The risk threshold of water is calculated by collecting the toxicity data of aquatic organisms with different statistical methods [18,19]. Predicted no-effect concentration (PNEC) is the ecological safety threshold proposed by the technical guidance document on risk assessment (TGD) [20]. PNEC is an important risk threshold of water for ecological risk assessment and management [21,22,23]. However, the studies about risk threshold in sediment was less than that in water. Researchers usually converted the concentration of pollutants in sediment to that in water; then, the risk of pollutants in sediment was analyzed based on the risk threshold of water [24,25,26]. In recent years, some researchers proposed to calculate PNEC in sediment (*PNEC_sediment_*), based on the Equilibrium Partitioning approach (EqP) [27]. However, this method failed to reflect the local characteristics of the water-sediment, because it was calculated only based on the octanol-water partition coefficient [28]. Therefore, it is critical to quantify the risk threshold for CAs in sediments scientifically.

Based on 23 sample data of CAs in the sediment of the Fenhe River Basin, the concentration and partitioning coefficients of CAs were investigated. Then, the *PNEC_sediment_* of CAs were calculated using the Equilibrium Partitioning approach and the partitioning coefficients. Finally, the risk was assessed using the risk quotients (*RQ*), based on the *PNEC_sediment_* calculation.

## 2. Sample Collection, Detection, and Analysis

### 2.1. Sample Collection and Detection

**Figure 1:**
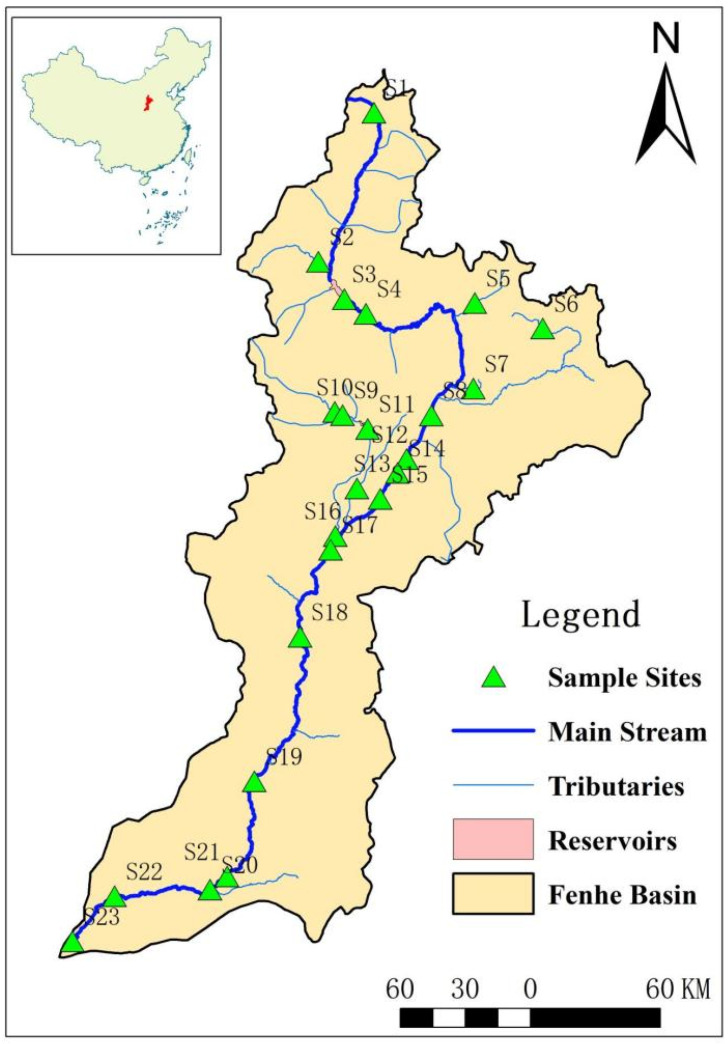
Sampling sites of the Fenhe River.

The Fenhe River is a tributary of the Yellow River in China and is located in the ecologically fragile area of the Loess Plateau, with a total length of 716 km and a basin area of 39,741 km^2^ (Figure 1). The Fenhe River is the main source of water for industrial and agricultural development in Shanxi Province. At the same time, the Fenhe River receives a large amount of sewage and wastewater [29]. Recently, researchers found that antibiotics were widely detected in the Fenhe River [9].

In this study, 23 samples were collected, covering the source, the major cities, reservoirs, intensive livestock and poultry breeding areas, the entrance of the Yellow River, and the main tributaries of the Fenhe River Basin in November 2019 (Figure 1). The sediment sample was collected using a Peterson mud collector. Approximately 1 kg sediment were collected into a 1 L brown glass bottle, in which 0.5 g of Na_2_-EDTA were added. All the samples were transported back to the laboratory immediately. In the laboratory, each sample was freeze-dried, then passed through a 2 mm sieve to remove the non-soil components to prevent the interference of biological matrix; then, each sample was passed through 60 mesh, making the samples much more uniform. The sample extracts were then analyzed by liquid chromatography tandem mass spectrometry, using an Agilent 1260–6460 Triple Quadrupole system (Agilent Technologies, Santa Clara, CA, USA). The LC-MS was equipped with electrospray ionization source (ESI) and MassHunter software, and Column was ZORBAX Eclipse Plus C18 column (1.8 μm × 2.1 mm × 50 mm I.D., Agilent, Santa Clara, CA, USA). The carrier flow rate was 0.4 mL/min, column temperature was 35 °C, ion source temperature was 320 °C, and scan mode was multiple reaction monitoring (Dynamic MRM). The mobile phase was water and acetonitrile. The targets contained three CAs, including Chloramphenicol (CHL), Thiamphenicol (THI), and Florfenicol (FF) (Table 1).

Calibration curves were constructed by analyzing standards containing all of the analytes, recovery standards, and internal standards (Table S1). The R^2^ of the calibration curves were >0.995, and the sediment analyses gave precision values (relative standard deviations) of 0.1–22.2% and accuracy values (recoveries) of 42–121%. The limits of detection were 0.01–7.89 µg/kg.

### 2.2. Partitioning Coefficients (Kp)

The partitioning coefficients (*Kp*) of CAs was the calculated concentration of CAs measured. The formula is shown by Equation (1):

(1) $$K_{p,w-s}=\frac{c_{s}}{c_{w}}$$

where, *K*_*p*,*w*-*s*_ is the partitioning coefficient used to characterize the water-sediment partition of pollutant; C_s_ is the concentration of CAs in the sediment phase (ng/g); and C_w_ is the concentration of CAs in the water phase (ng/L).

### 2.3. Predicted No-Effect Concentration

The predicted no-effect concentration (PNEC) of antibiotics in the sediment was calculated using the Equilibrium Partitioning approach (EqP), which was recommended by the technical guidance document on risk assessment [30]. The formula is shown by Equations (2) and (3):

(2) $$PNEC_{sediment}=\frac{K_{p,w-s}}{RHO_{sediment}}\times PNEC_{water}\times 1000$$

(3) $$RHO_{sediment}=F_{solid}\times RHO_{solid}+F_{water}\times RHO_{water}$$

where, *PNEC_sediment_* is the predicted no-effect concentration of CAs in sediment (µg/L); *RHO_sediment_* is the bulk density of sediment (kg/m^3^); *F_water_* is the volume fraction of the water phase; *F_solid_* is the volume fraction of the sediment phase; *RHO_solid_* is the approximated density of the solid phase (kg/m^3^); and *RHO_water_* is the approximated density of the water phase (1000 kg/m^3^).

### 2.4. Ecological Risk Assessment

Taking the *PNEC_sediment_* as the risk threshold, the ecological risk of the CAs in sediment was assessed using risk quotients (*RQ*). Risk quotients were classified into four levels: <0.01, insignificant risk; 0.01–0.1, low-risk; 0.1–1, medium-risk; and >1, high-risk. The *RQ* was calculated using the following equation [26]:

(4) $$RQ=\frac{C}{PNEC_{sediment}}$$

where, *RQ* is the risk of CAs; and *C* is the concentration of detected CAs (µg/L).

## 3. Results and Discussion

### 3.1. Concentration of CAs in Sediment and Temporal Variations

**Figure 2:**
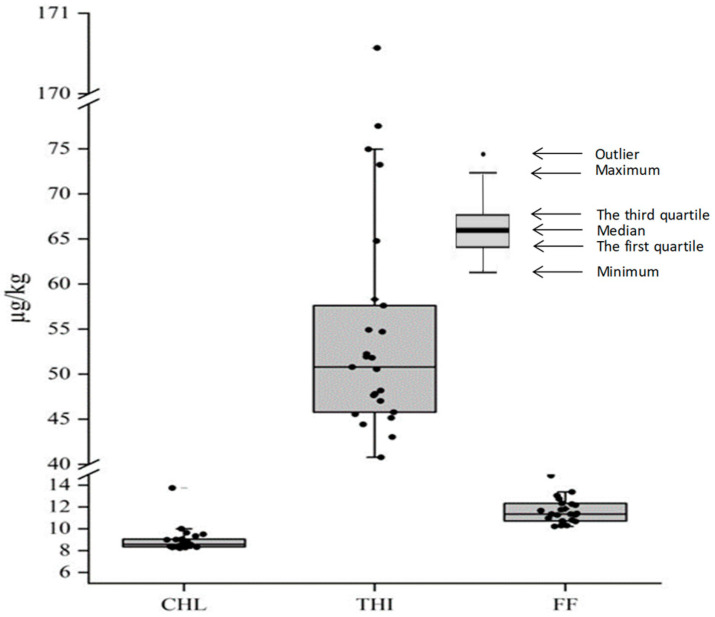
Concentration of CAs in sediments of the Fenhe River.

All CAs were detected in the sediments of the Fenhe River Basin, and the mean concentration was 79.1 µg/kg (Figure 2). The concentrations of these three CAs were THI > FF > CHL, and CAs were detected in all the samples. The concentration of THI was obvious among these three CAs, and the mean concentration was 58.3 µg/kg. The concentrations of FF and CHL were relatively lower than that of THI, which were 11.8 µg/kg and 8.97 µg/kg, respectively. The variability of CHL and FF were less in the basin, and the standard deviations were 1.15–1.60, while the standard deviation of THI was higher than 26.5, which directly led to the standard deviation of CAs as high as 26.2.

**Figure 3:**
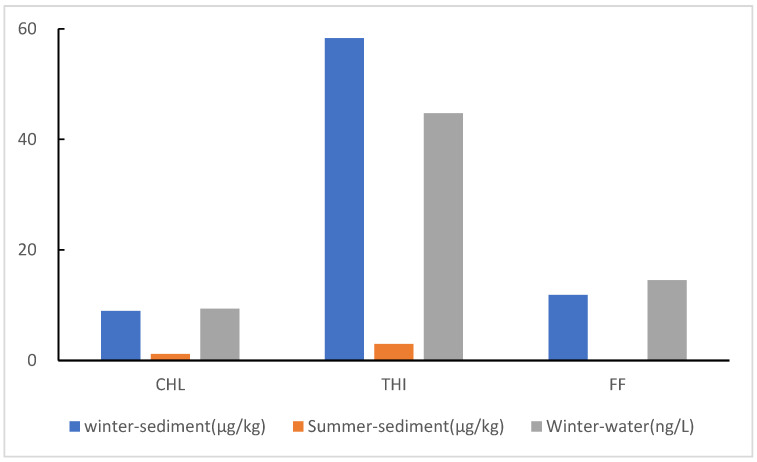
The concentration of CAs in different seasons and phases.

Temporally, the results showed that the concentrations of CAs in the sediments were different in different seasons (Figure 3). The concentration of CAs was higher in winter than that in summer, up to 4.79–174 times [31]. Similarly, THI was the dominant antibiotic in different seasons, with a concentration range from 2.15 μg/kg to 9.40 μg/kg and a mean concentration of 2.97 μg/kg in summer, much lower than the concentration in this study. The CHL was also detected in the whole basin in summer, but the concentration ranged from 0.52 μg/kg to 11.4 μg/kg, and the mean concentration was 1.17 μg/kg, which was also much lower than that in this study [31]. Moreover, FF was not detected in the whole basin in summer. The concentration of CAs in S8 in the main stream was the highest among all the sample sites, indicating that the concentrated discharge of domestic sewage and aquaculture wastewater in the Taiyuan and Jinzhong areas caused the extremely high concentration [9].

### 3.2. Spatial Distribution of CAs in Sediment

**Figure 4:**
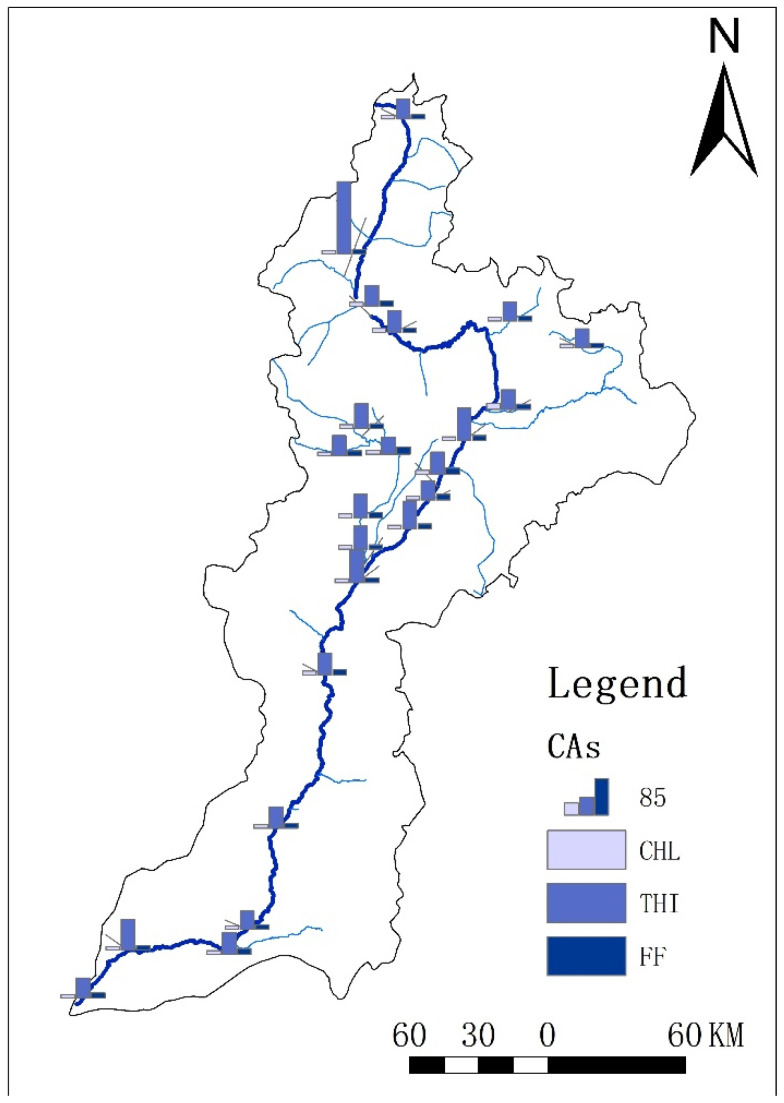
Spatial variation of concentration for CAs in sediments of the Fenhe River (the column means the concentration of CAs and the color means different antibiotics).

Spatially, the mean concentration of CAs in the midstream was 83.5 μg/kg, which was higher than that in the upstream (mean, 73.3 μg/kg) and downstream (mean, 68.0 μg/kg) (Figure 4). The concentration proportion of THI was as high as 75.4% in the midstream of the Fenhe River Basin, and the proportions in the upstream and downstream were 72.3% and 72.0%, respectively. However, it was slightly different in summer. In the summer, the concentration of CAs was highest in the downstream, and the mean concentration was 3.80 μg/kg. Compared with the concentration of CAs in the downstream, the concentration of CAs in the midstream was slightly lower (mean, 3.75 μg/kg), and that in the upstream was the lowest (mean, 2.97 μg/kg).

The concentrations of CAs in S8 and S17 in the midstream were relatively prominent, as high as 93.8 μg/kg and 97.8 μg/kg, respectively. They were at the exit of the Taiyuan section in the midstream of the Fenhe River. The S22 was at the exit of the Fenhe River Basin, and the concentration of CAs was also as high as 93.0 μg/kg. However, the highest concentration of CAs in sediments in the main stream in the summer were distributed in S21 and S17, and the concentrations were 7.29 μg/kg and 5.67 μg/kg, respectively [31].

The concentration of CAs in tributaries were generally higher than that in the main stream, and the mean concentration was 83.0 μg/kg. The concentration of THI also accounted for a higher proportion than that of others; the proportion was 73.9%. The concentration of CAs in S2 was the most prominent in the tributaries, with the concentration being up to 190.8 μg/kg, the highest concentration in the whole basin. The concentration of THI in S2 was also the highest, which was 170.6 μg/kg, while the concentration of CAs in other tributaries ranged from 65.2 μg/kg to 77.1 μg/kg, and the variation of concentration was relatively small. Similar to the results in this study, the concentration of CAs in the tributaries was also higher than that in the main stream in summer in the Fenhe River Basin, with a mean concentration of 4.85 μg/kg. However, the highest concentration was distributed in S6, which lay in the upstream of the Xiaohe River, with a concentration up to 13.7 μg/kg, followed by S2, in the Lanhe River, whose concentration was 9.94 μg/kg. The difference of concentration in other tributaries was small, with a range of 2.91–3.38 μg/kg.

### 3.3. Analysis of Risk Threshold for CAs

Combined with the results of CAs in water [9], the distribution coefficient (*Kp*) of the CAs was calculated. The results showed that *Kp* of CAs ranged from 865 L/kg to 21,328 L/kg, among which the *Kp* of THI was the highest, and the range was 819–4201 L/kg (Table 2).

**Figure 5:**
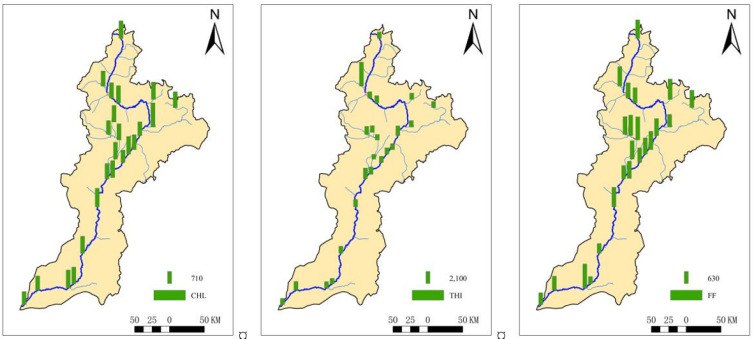
Spatial distribution of *Kp* for CAs of the Fenhe River (the column means the value of the *Kp*).

Spatially, the *Kp* of CAs in the midstream was 876 L/kg, which was lower than that in the upstream (988 L/kg) and downstream (939 L/kg) (Figure 5). Among them, the highest *Kp* was THI at S2, as high as 4201 L/kg, which was located in the downstream of Lan County. Compared with the *Kp* of CAs in the main stream, the *Kp*s of CAs in the tributaries were generally higher, and the average *Kp* was as high as 1011 L/kg.

By testing the sediment samples, the range of *F_water_* in the Fenhe River Basin was 0.499–0.554 (mean, 0.531), the range of *F_sediment_* was 0.446–0.501 (mean, 0.469), and the range of *RHO_solid_* was 1580–1970 kg/m^3^ (mean, 1796 kg/m^3^) (Table 3). Compared with that of other rivers, the *F_water_* in the Fenhe River Basin was relatively lower, and the value of *RHO_solid_* in the Fenhe River Basin was significantly higher [32]. The difference of *F_water_*, *F_sediment_*, and *RHO_solid_* in the Fenhe River Basin was small in the whole basin, and the coefficient of variation was not higher than 0.051. Spatially, the differences of *F_water_*, *F_sediment_*, and *RHO_solid_* in the upstream, midstream, and downstream were also very small; the coefficient of variation was less than 0.008. Relatively speaking, the difference of *F_water_*, *F_sediment_*, and *RHO_solid_* in the tributary and the main stream was large; the coefficient of variation was 1.9–2.3 times that of the main stream. Based on the data of *F_water_*, *F_sediment_*, and *RHO_solid_*, *RHO_sediment_* was calculated. The results showed that the range of *F_sediment_* was 1290–1432 kg/m^3^ in the whole basin, and the coefficient of variation was only 0.024.

Using the toxicity data EC_50_ collected from the available literatures [33,34,35], the *PNEC_water_* and the *PNEC_sediment_* was calculated (Table 3). The results showed that the values of risk threshold for Cas in sediment were 56.3–251.8 mg/kg. Comparing with previous risk studies, researchers usually converted antibiotic concentrations in sediment to concentrations in water, due to the lack of toxicity data in sediment; then, the risk was assessed based on the risk threshold of CAs in the water, which cannot reflect the local characteristics of the water and sediment [24,25,26,28].

### 3.4. Risk Assessment of Chloramphenicol Antibiotics of Sediment

**Figure 6:**
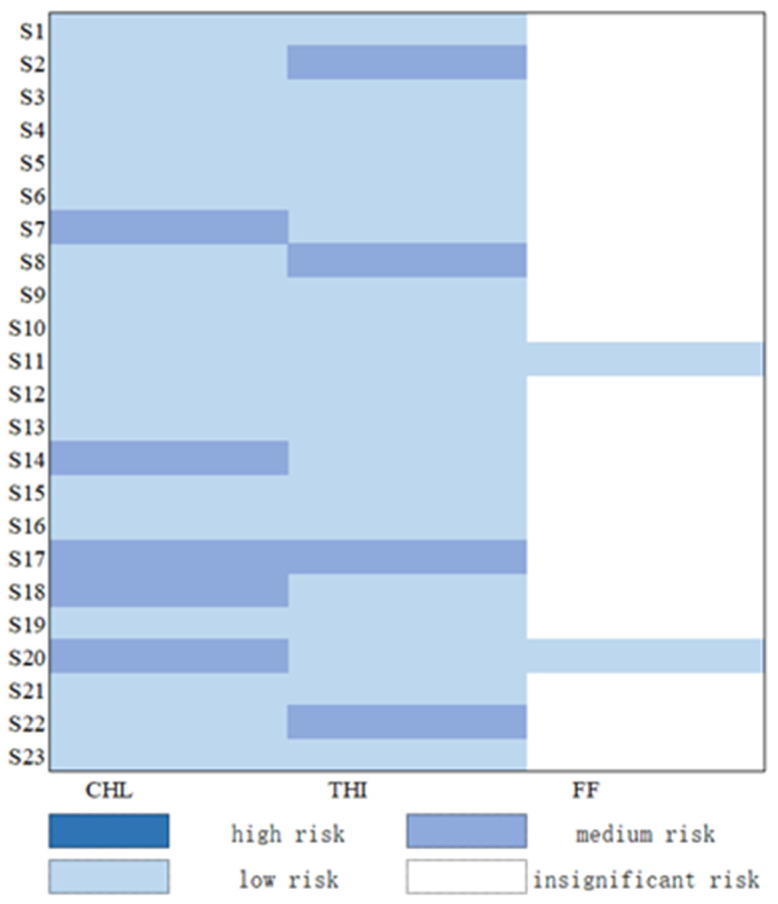
Risk assessment of CAs in sediment of the Fenhe River (the *Y*-axis means different sample sites and *X*-axis means different antibiotics).

Taking the calculated *PNEC_sediment_* as the risk threshold, the ecological risk of CAs in sediment in the Fenhe River Basin was assessed (Figure 6). The results showed that the risk area of CHL was the biggest among these three CAs. All the areas showed different risk levels in the whole basin, with the area proportion of medium-risk level accounting for 21.7%. Besides, all the areas also showed different risk levels for THI in the whole basin, and the area proportion of medium-risk level was 17.4%. Different to CHL and THI, the risk area of FF was the lowest; only 8.7% of the area showed a low-risk level in the whole basin.

The sites which showed medium risk for CHL were distributed in the midstream and downstream of the Fenhe River Basin, including S14 (Qi County), S17 (Yitang County), and S18 (Wangzhuang Bridge South). The sites S7 (Taiyu drainage canal) and S20 (Huihe River) in tributaries also showed medium risk for CHL. The sites which showed medium risk for THI were also distributed in the midstream, downstream, and tributaries, but only site S17 was overlapped with CHL. In addition, the sites S8 (WennanShe) and S22 (Hejin Bridge), in the main stream, and the site S2 (Lanhe River), in the tributaries, also showed medium risk for THI. For FF, there were only two sites that showed low risk, S11 (Wenyu River) and S20 (Hui River), located in the tributaries. Frequent human activities in the midstream had a great impact on the concentration of the CAs [37,38].

## 4. Discussion

The results showed that the concentrations of CAs in the sediments were different in different seasons. The reason for temporal variation might be that lower water temperature, weaker solar radiation, and smaller flow rate in winter leads to lower degradation ratio of chloramphenicol antibiotics and easier access to sediment [39,40,41]. Compared with previous studies about CAs in water, the concentration in sediment was higher; the reason for this might be that CA was easily adsorbed in the suspended matter and, finally, existed in sediment [9,42,43,44]. As the second tributary of the Yellow River, the sand in the Fenhe River Basin is higher than that in other rivers. The suspended matter is more likely to carry CAs into the sediment [22]. The lower water temperature and slower water flow in winter also leads to the lipophilic antibiotics accumulated in the sediment [45].

Spatially, the mean concentration of CAs in the midstream was higher than that in the upstream and downstream. Mountainous areas and less human activity in the upstream might result in less antibiotic residue than that in the midstream and downstream [46]. The higher concentration of CAs in the midstream and downstream might be the reason that these regions were dominated by some large- and medium-sized cities, where there are distributed many industries [47,48]. Combined with the measured results of CAs in water [9], it is indicated that the delayed deposition effect for CAs occurred in the midstream and downstream of the Fenhe River Basin in winter. With the water flowing to the downstream, more antibiotic substances were carried into the downstream, gradually entering and accumulating in the sediment [49,50]. Compared to the concentration of CAs in summer, the degradation efficiency and the dilution effect of Cas was weaker in winter, with the lower temperature and the smaller water flow [25,45].

Compared with the *Kp* of CAs in summer [9], the *Kp* of CAs in winter was significantly higher, probably because the water temperature in summer is higher, and it was, therefore, easy for the CAs to experience hydrolysis and photolysis in the water [41]. Besides, the difference of flow in summer and winter might be another reason [31]. The flow of water was smaller in winter, so the CAs had enough time to exchange into the sediment, leading to the higher concentration of CAs in the sediment [9,45,51,52]. Spatially, the *Kp* of CAs in the midstream was lower than that in the upstream and downstream. The reason might be that the flow rate of the midstream was fast, so that a large number of CAs were washed to the downstream and, finally, accumulated in the sediment [53]. Conversely, the flow in the tributaries was slower than that in the main stream; CAs can exchange adequately from water to sediment, resulting in a higher *Kp* value.

In the field of risk assessment for antibiotics in sediment, previous studies calculated sediment risk only based on the octanol-water coefficient (kow), while the regional ratio of water to sediment was not considered [54,55]. The ecological risk assessment of sediment based on the water risk threshold has some disadvantages [30,56]. Based on the measured data, this study calculated the risk threshold in sediment using the EqP method to reflect the physical and chemical characteristics of the basin, obtaining a more accurate risk threshold. The EqP method is based on non-ionic organic chemicals in sediments, and is used to predict bioavailable material components in soil pore water [57]. Moreover, the volume ratio of water-sediment has a great influence on the calculation process of the risk threshold [30]. The concentration of suspended mater in the Yellow River Basin is usually higher than that in other rivers, resulting in a low volume fraction of the water phase [58]. Therefore, it is more scientific and reliable to calculate the sediment risk threshold using the EqP based on the measured data in the watershed environment [27,59].

## 5. Conclusions

Based on 23 sample data of CAs in sediment in the Fenhe River Basin, the concentration and risk of CAs were investigated. The results showed that CAs were detected in all the samples in the sediments in the Fenhe River Basin, and the mean concentration was 79.1 μg/kg. The concentration of THI was obvious among these three CAs, and the mean concentration was 58.3 µg/kg.

Based on the measured data of Cas in the water and sediment, the *Kp*, the *F_water_*, *F_sediment_*, and *RHO_solid_* were calculated in the Fenhe River Basin. Then, the *PNEC_sediment_* was calculated; the results were 56.3–251.8 mg/kg. Based on the calculated *PNEC_sediment_*, the ecological risk of CAs in sediment in the Fenhe River Basin was assessed. The results showed that the risk area of CHL was the biggest among these three CAs, all the areas showed different risk levels in the whole basin, and the area proportion of medium-risk level accounted for 21.7%. Besides, all the areas also showed different risk levels for THI in the whole basin, and the area proportion of medium-risk level were 17.4%. Different to CHL and THI, the risk area of FF was the lowest; only 8.7% of the area showed a low-risk level in the whole basin.

## Figures and Tables

**Table 1:** The classification of antibiotics.

Antibiotics		Molecular Formula	CAS	Molecular Weight (g/mol)
Chloramphenicol	CHL	C_11_H_12_Cl_2_FN_2_O_5_	154–75–2	323.1
Thiamphenicol	THI	C_12_H_15_C_l2_NO_5_S	15,318–45–3	356.2
Florfenicol	FF	C_12_H_14_Cl_2_FNO_4_S	73,231–34–2	358.2

**Table 2:** Mean of sediment-water partitioning coefficient of antibiotics.

Antibiotics	Summer (L/kg)	Winter (L/kg)
CHL	294	963
THI	188	2972
FF	-	865

**Table 3:** The risk threshold reference for CAs in water and sediment.

Antibiotics	Most Sensitive Species	*EC*_50_ (mg/L)	*PNEC_water_* (mg/L)	*PNEC_sediment_* (mg/kg)
CHL	*Desmodesmus subspicatus*	0.13 [33]	130	113.6
THI	*Microcystis aeruginosa*	0.32 [36]	320	56.3
FF	*Pseudokirchneriella subcapitata*	2.30 [35]	2300	251.8

## Data Availability

All the data used to support the findings of this study are available from the corresponding author upon request.

## Supplementary Materials

The following are available online at https://www.mdpi.com/article/10.3390/toxics11070570/s1, Table S1: Information of quality control of sample detecting.
