# Supplementary material for: Risk Threshold and Assessment of Chloramphenicol Antibiotics in Sediment in the Fenhe River Basin, China
Source: Toxics. 2023 Jun 30;11(7):570. doi: 10.3390/toxics11070570 (PMC10385513; doi:10.3390/toxics11070570)
Supplement: Supplementary file 1 [file toxics-11-00570-s001.zip › toxics-2437681-supplementary.pdf]

toxics

Supplementary Materials

Risk Threshold and Assessment of Chloramphenicol Antibiotics in Sediment in the Fenhe River Basin, China

Linfang Wang [1,*], Dexuan Dang [1], Leiping Cao [2], Huiyan Wang [1] and Ruimin Liu [2]

[1] Shanxi Key Laboratory of Sorghum Genetic and Germplasm Innovation, Sorghum Research Institute, Shanxi Agricultural University, Jinzhong 030600, China
[2] State Key Laboratory of Water Environment Simulation, School of Environment, Beijing Normal University, Beijing 100875, China
* Correspondence: wanglfsx@foxmail.com; Tel.: +86-35-4859-3516

Table S1. The information of quality control of sample detecting.

Name	Standard Curve Equation	R^2	Precision %	Accuracy %	Limits of Detection µg/kg
CHL	Y=1.133313X-0.057702	0.9952	16.8	84.3	2.06
THI	Y=0.393087X-0.083158	0.9964	3.1	58.5	4.76
FF	Y=1.707616X+0.115526	0.9952	0.1	84.3	1.58
